# Type 2 Diabetes, Antidiabetic Medications, and Colorectal Cancer Risk: Two Case–Control Studies from Italy and Spain

**DOI:** 10.3389/fonc.2016.00210

**Published:** 2016-10-06

**Authors:** Valentina Rosato, Alessandra Tavani, Esther Gracia-Lavedan, Elisabet Guinó, Gemma Castaño-Vinyals, Cristina M. Villanueva, Manolis Kogevinas, Jerry Polesel, Diego Serraino, Federica E. Pisa, Fabio Barbone, Victor Moreno, Carlo La Vecchia, Cristina Bosetti

**Affiliations:** ^1^Department of Epidemiology, IRCCS − Istituto di Ricerche Farmacologiche Mario Negri, Milan, Italy; ^2^ISGlobal, Centre for Research in Environmental Epidemiology (CREAL), Barcelona, Spain; ^3^Universitat Pompeu Fabra (UPF), Barcelona, Spain; ^4^CIBER Epidemiología y Salud Pública (CIBERESP), Madrid, Spain; ^5^Cancer Prevention and Control Program, Unit of Biomarkers and Susceptibility, Catalan Institute of Oncology (ICO)-IDIBELL, Hospitalet de Llobregat, Barcelona, Spain; ^6^Hospital del Mar Medical Research Institute (IMIM), Barcelona, Spain; ^7^Unit of Cancer Epidemiology, CRO Aviano National Cancer Institute, IRCCS, Aviano, Italy; ^8^SOC Igiene ed Epidemiologia Clinica, Azienda Ospedaliero Universitaria di Udine, Udine, Italy; ^9^Department of Medical and Biological Sciences, University of Udine, Udine, Italy; ^10^Department of Clinical Sciences, Faculty of Medicine, University of Barcelona, Barcelona, Spain; ^11^Department of Clinical Sciences and Community Health, University of Milan, Milan, Italy

**Keywords:** antidiabetic medications, colorectal cancer, diabetes, insulin, metformin, risk factor

## Abstract

**Background:**

Type 2 diabetes mellitus has been associated with an excess risk of colorectal cancer, although the time–risk relationship is unclear, and there is limited information on the role of antidiabetic medications.

**Aim:**

We examined the association between type 2 diabetes, antidiabetic medications, and the risk of colorectal cancer, considering also duration of exposures.

**Methods:**

We analyzed data derived from two companion case–control studies conducted in Italy and Spain between 2007 and 2013 on 1,147 histologically confirmed colorectal cancer cases and 1,594 corresponding controls. Odds ratios (ORs) and 95% confidence intervals (CIs) were estimated by unconditional multiple logistic regression models, adjusted for socioeconomic factors and major potential confounding factors.

**Results:**

Overall, 14% of cases and 12% of controls reported a diagnosis of diabetes, corresponding to an OR of colorectal cancer of 1.21 (95% CI 0.95–1.55). The OR was 1.49 (95% CI 0.97–2.29) for a duration of diabetes of at least 15 years. The OR was 1.53 (95% CI 1.06–2.19) for proximal colon cancer, 0.94 (95% CI 0.66–1.36) for distal colon cancer, and 1.32 (95% CI 0.94–1.87) for rectal cancer. In comparison with no use, metformin use was associated with a decreased colorectal cancer risk (OR 0.47, 95% CI 0.24–0.92), while insulin use was associated with an increased risk (OR 2.20, 95% CI 1.12–4.33); these associations were stronger for longer use (OR 0.36 and 8.18 for ≥10 years of use of metformin and insulin, respectively).

**Conclusion:**

This study shows evidence of a positive association between diabetes and colorectal cancer, mainly proximal colon cancer. Moreover, it indicates a negative association between colorectal cancer and metformin use and a positive association for insulin use.

## Introduction

Colorectal cancer is the third most common cancer and the fourth leading cause of cancer death worldwide, and the fourth and second, respectively, in Europe (1, 2). Among established risk factors for this neoplasm are high consumption of red and processed meat, heavy alcohol consumption, body fatness, and family history of colorectal cancer, whereas physical activity, regular aspirin use, statin use, and, probably, a diet rich in fiber, especially from fruit and vegetables, appear to have a protective role against this neoplasm (3–6).

Type 2 diabetes mellitus has been associated with an excess risk of colorectal cancer (7–10), as well as with of cancers related to metabolic factors (11, 12). Although the magnitude of the excess risk appears to be modest (about 30%), it may have important public health implications given the high prevalence of diabetes. The association has been found to be consistent between study designs (i.e., cohort versus case–control studies), sex, and cancer subtypes (i.e., colon versus rectum) (7, 9, 13, 14). However, only a few studies considered the timing of the disease in relation to colorectal cancer diagnosis, reporting no clear trend in risk (9).

Diabetes medications have also been associated with colorectal cancer risk in some studies which suggested that insulin use may increase the risk (15), while metformin (15–17) and thiazolidinedione (15, 18) use may reduce it. The evidence on diabetes medications is, however, still inconsistent, and only scanty studies considered time–risk relationships (15).

We further examined the association between type 2 diabetes, antidiabetic medications, and colorectal cancer risk using data from two companion case–control studies from Italy and Spain, where information on duration of exposures was considered.

## Materials and Methods

### Study Population

The present data derive from the HIWATE Project (19, 20), which includes two companion case–control studies conducted in Italy and Spain between September 2007 and December 2013. The first was conducted in Northern Italy (Milan and Pordenone/Udine areas) between 2008 and 2010 on 456 colorectal cancer cases (median age 67, range 35–80) and 569 controls (median age 66, range 31–80 years); the latter was conducted in Spain (Barcelona area) between 2007 and 2013 on 696 colorectal cancer cases (median age 68, range 22–85) and 1,036 controls (median age 66, range 28–85 years). Patients with no information on diabetes history (two cases and six controls), with a diagnosis of type 1 diabetes (one case and one control from Italy), or with a diagnosis of diabetes before age 30 (two cases and four controls) were excluded from the present analyses, thus leaving a total of 1,147 cases and 1,594 controls, 455 and 567 from Italy and 692 and 1,027 from Spain, respectively.

In both studies, cases were incident, histologically confirmed colorectal cancer patients, admitted to reference study centers in the study areas. Three hundred nine cases had a diagnosis of proximal colon cancer (i.e., cecum, International Classification of Diseases, vol. 10, ICD 10 = C18.0, ascending colon, ICD 10 = C18.2, hepatic flexure, ICD 10 = C18.3, or transverse colon, ICD 10 = C18.4), 415 of distal colon cancer (i.e., splenic flexure, ICD 10 = C18.5, descending colon, ICD 10 = C18.6, or sigmoid colon, ICD 10 = C18.7), 16 of overlapping colon cancer or not otherwise specified colon (NOS) cancer, ICD 10 = C18.8 and C18.9, 397 of rectal cancer (i.e., rectosigmoid junction, ICD 10 = C19.9, or rectum, ICD 10 = C20.9) and for 10 cases the anatomical subsite was not indicated. In Italy, controls were patients admitted to the same hospitals as cases for a wide spectrum of acute, non-neoplastic conditions unrelated to factors likely related to colorectal cancer. In Spain, controls were population-based and were identified from the lists of selected family practitioners and contacted by telephone. Cases and controls were resident in the enrollment areas for at least 6 months prior to diagnosis (cases) or recruitment (controls). The study protocols were approved by the ethical review boards of the participating centers, and all participants signed an informed consent form before recruitment. Average response rates among patients approached were 95% in Italy and 68% in Spain, among cases, and 95% in Italy and 53% in Spain, among controls.

All patients were interviewed by *ad hoc* trained interviewers using similar structured questionnaires to collect personal information on sociodemographic factors, lifestyle habits (e.g., tobacco smoking, alcohol drinking, physical activity, and dietary habits), anthropometric measures, a problem-oriented medical history, and family history of cancer. In both studies, history of diabetes was self-reported and included the age at first diagnosis. In the Spanish study, additional information on antidiabetic medications was collected, including use of oral antidiabetic drugs and insulin, and corresponding duration. Only patients with treated diabetes and a diagnosis of diabetes more than 1 year before cancer diagnosis/interview were considered among diabetic patients.

### Statistical Analysis

To assess the association between diabetes and diabetes-related variables and colorectal cancer risk, we used unconditional logistic regression to estimate the odds ratios (ORs) and corresponding 95% confidence intervals (CIs) adjusted for sociodemographic factors (i.e., study center, sex, age, and education) and major potential confounding factors, i.e., tobacco smoking, alcohol drinking, body mass index, lifetime leisure physical activity, statin use, and regular aspirin use. Further adjustment for consumption of meat, fruit, and vegetables and for total energy intake was also considered. In sensitivity analyses, we provided the OR for patients with a diagnosis of diabetes more than 2 or more years (or 3 or more years) prior to cancer diagnosis.

To assess the association between colorectal cancer and antidiabetic medications, we selected diabetic patients only. Therefore, the risk of colorectal cancer for diabetic patients using a specific antidiabetic drug was compared with that of diabetic patients using other antidiabetic medications. Alternatively, we considered as reference category diabetic patients using other antidiabetic medications plus non-diabetic patients.

We used additional models to assess the potential modifying effect of selected covariates on the association with diabetes and colorectal cancer risk, and we tested for heterogeneity across strata of the covariates (i.e., multiplicative interaction) using likelihood ratio tests and the resulting χ^2^ statistics. All statistical analyses were performed with SAS 9.2 statistical software (SAS Institute, Cary, NC, USA).

## Results

Table 1 gives the distribution of colorectal cancer cases and controls, according to selected characteristics. Cases and controls had a similar distribution by sex, tobacco smoking, alcohol drinking, body mass index, leisure-time physical activity, and regular aspirin use. Cases were somewhat older and heavier alcohol drinkers, and reported a lower level of education, and a lower use of statins than controls.

**Table 1:** **Distribution of 1,147 colorectal cancer cases and 1,594 controls, according to sex, age, and other selected characteristics**.

Characteristics	Cases		Controls		*p*-Value^a^
	*N*	%	*N*	%	
**Study center**					
Milan, Italy	238	20.7	247	15.5	
Pordenone/Udine, Italy	217	18.9	320	20.1	
Barcelona, Spain	692	60.3	1,027	64.4	0.002
**Sex**					
Men	750	65.4	998	62.6	
Women	397	34.6	596	37.4	0.14
**Age**					
<60	259	22.6	428	26.9	
60–64	179	15.6	266	16.7	
65–69	236	20.6	318	19.9	
70–74	211	18.4	293	18.4	
≥75	262	22.8	289	18.1	0.0008
**Education (years)**					
<8	676	58.9	790	49.6	
8–12	286	24.9	456	28.6	
≥13	183	16.0	347	21.8	<0.0001
Missing	2	0.2	1	0.1	
**Tobacco smoking**					
Never smokers	459	40.0	664	41.7	
Former smokers	467	40.7	618	38.8	
Current	214	18.7	310	19.4	0.53
Missing	7	0.6	2	0.1	
**Alcohol consumption (drinks/day)**					
0	271	23.6	265	16.6	
>0 to <1	274	23.9	535	33.6	
1 to <4	359	31.3	517	32.4	
≥4	183	16.0	160	10.0	0.43
Missing	60	5.2	117	7.3	
**Body mass index (kg/m^2^)**					
<25	399	34.8	544	34.1	
25 to <30	505	44.0	706	44.3	
≥30	242	21.1	340	21.3	0.76
Missing	1	0.1	4	0.3	
**Physical activity (METS h/week)**					
0	287	25.0	362	22.7	
>0 to 8	388	33.8	546	34.3	
>8	472	41.2	648	40.7	0.46
Missing	0	0	38	2.4	
**Statin use**					
Never	909	79.3	1,144	71.8	
Ever	233	20.3	448	28.1	<0.0001
Missing	5	0.4	2	0.1	
**Regular aspirin use**					
Never	977	85.2	1,336	83.8	
Ever	157	13.7	250	15.7	0.15
Missing	13	1.1	8	0.5	

*Italy and Spain, 2007–2013*.

*METS, metabolic equivalents of task*.

*^a^*p*-Values calculated from χ^2^ statistics (χ^2^ for trend for ordinal variables)*.

The distribution of colorectal cancer cases and controls by history of diabetes and the corresponding ORs by country and overall are shown in Table 2. Overall, 159 (13.9%) of cases and 188 (11.8%) of controls reported a diagnosis of type 2 diabetes, corresponding to a multivariable OR of 1.21 (95% CI 0.95–1.55). The OR was 1.12 in the Italian study and 1.20 in the Spanish study. The OR of colorectal cancer was higher for a duration of diabetes of ≥15 years (OR 1.49, 95% CI 0.97–2.29), but the trend in risk for increasing duration was not significant (*p*-value = 0.10). Corresponding values were 1.68 in Italy and 1.47 in Spain. In sensitivities analyses excluding patients with a diagnosis of diabetes within 2 or 3 years prior to cancer diagnosis, the OR were 1.16 (95% CI 0.89–1.50) and 1.15 (95% CI 0.88–1.50), respectively, in the two studies combined (data not shown). Further allowance for selected dietary factors (e.g., meat, fruit or vegetables) or total energy intake did not meaningfully change our risk estimates (data not shown).

**Table 2:** **Distribution of 1,147 colorectal cancer cases and corresponding 1,594 controls, with odds ratios (ORs) and 95% confidence intervals (CIs), according to history of diabetes by country and overall**.

	Italy			Spain			Overall		
	Cases *N* (%)	Controls *N* (%)	OR^a^ (95% CI)	Cases *N* (%)	Controls *N* (%)	OR^a^ (95% CI)	Cases *N* (%)	Controls *N* (%)	OR^a^ (95% CI)
**History of diabetes**									
No	414 (91.0)	520 (91.7)	1.00^b^	574 (82.9)	886 (86.3)	1.00^b^	988 (86.1)	1,406 (88.2)	1.00^b^
Yes	41 (9.0)	47 (8.3)	1.12 (0.70–1.78)	118 (17.1)	141 (13.7)	1.20 (0.89–1.62)	159 (13.9)	188 (11.8)	1.21 (0.95–1.55)
**Duration of diabetes (years)**									
1 to <5	11 (26.8)	14 (29.8)	1.05 (0.45–2.45)	32 (27.1)	34 (24.1)	1.31 (0.77–2.24)	43 (27.0)	48 (25.5)	1.21 (0.78–1.88)
5 to <10	7 (17.1)	7 (14.9)	1.18 (0.39–3.57)	22 (18.6)	39 (27.7)	0.85 (0.48–1.50)	29 (18.2)	46 (24.5)	0.96 (0.59–1.57)
10 to <15	6 (14.6)	13 (27.7)	0.58 (0.21–1.62)	25 (21.2)	28 (19.9)	1.20 (0.66–2.18)	31 (19.5)	41 (21.8)	1.12 (0.68–1.83)
≥15	17 (41.5)	13 (27.7)	1.68 (0.78–3.62)	31 (26.3)	34 (24.1)	1.47 (0.86–2.51)	48 (30.2)	47 (25.0)	1.49 (0.97–2.29)
Missing	–	–		8 (6.8)	6 (4.3)		8 (5.0)	6 (3.2)	
*p*-Value for trend			0.49			0.20			0.10

*Italy and Spain, 2007–2013*.

*^a^Estimates from multiple logistic regression models including terms for study center, sex, age, education, tobacco smoking, alcohol drinking, body mass index, physical activity, statin use, and aspirin use*.

*^b^Reference category*.

No significant heterogeneity was observed in risk estimates across strata of sex, age, education, tobacco smoking, alcohol consumption, body mass index, physical activity, and statin and aspirin use (Table S1 in Supplementary Material).

Table 3 shows the distribution of anatomical subsites of colorectal cancer cases and corresponding controls, and the OR estimates according to history of diabetes by country and overall. In both countries combined, the OR was 1.15 (95% CI 0.89–1.55) for colon cancer overall, 1.53 (95% CI 1.06–2.19) for proximal colon, 0.94 (95% CI 0.66–1.36) for distal colon, and 1.32 (95% CI 0.94–1.87) for rectal cancer. Corresponding estimates were 1.04, 1.32, 0.94, and 1.46 in Italy and 1.15, 1.64, 0.88, 1.27 in Spain. The OR for overlapping and NOS colon cancer was not shown due to the limited number of patients.

**Table 3:** **Distribution of anatomical subsites of colorectal cancer^a^ cases and corresponding controls, with odds ratios (ORs) and 95% confidence intervals (CIs), according to history of diabetes by country and overall**.

History of diabetes	Italy		Spain		Overall	
	Cases/controls	OR^b^ (95% CI)	Cases/controls	OR^b^ (95% CI)	Cases/controls	OR^b^ (95% CI)
**Overall colon cancers^a^**						
No	274/520	1.00^c^	365/886	1.00^c^	639/1,406	1.00^c^
Yes	26/47	1.04 (0.61–1.76)	75/141	1.15 (0.82–1.63)	101/188	1.15 (0.89–1.55)
**Proximal colon cancers**						
No	109/520	1.00^c^	147/886	1.00^c^	256/1,406	1.00^c^
Yes	13/47	1.32 (0.67–2.62)	40/141	1.64 (1.05–2.56)	53/188	1.53 (1.06–2.19)
**Distal colon cancers**						
No	150/520	1.00^c^	218/886	1.00^c^	368/1,406	1.00^c^
Yes	12/47	0.94 (0.46–1.88)	35/141	0.88 (0.57–1.36)	47/188	0.94 (0.66–1.36)
**Rectal cancers**						
No	140/520	1.00^c^	200/886	1.00^c^	340/1,406	1.00^c^
Yes	15/47	1.46 (0.74–2.85)	42/141	1.27 (0.84–1.93)	57/188	1.32 (0.94–1.87)

*Italy and Spain, 2007–2013*.

*^a^These include proximal colon, distal colon, and overlapping or not otherwise specified colon cancers*.

*^b^Estimates from multiple logistic regression models including terms for study center, sex, age, education, tobacco smoking, alcohol drinking, body mass index, physical activity, statin use, and aspirin use*.

*^c^Reference category, no history of diabetes*.

Table 4 shows the distribution of colorectal cancer cases and controls, and the corresponding ORs, according to use of antidiabetic medications in diabetic patients from the Spanish study. Diabetic patients using any oral antidiabetic drug had a non-significant reduced risk of colorectal cancer as compared to those not using them (OR 0.59, 95% CI 0.18–1.92). The OR was significantly below unity in diabetic patients using metformin (OR 0.47, 95% CI 0.24–0.92) as compared with those not using it, with a trend of decreasing risk with increasing duration of use (OR 0.36, 95% CI 0.15–0.85, for ≥10 years of use; *p*-value for trend 0.005). Among other oral antidiabetic drugs, sulfonylurea derivatives were not significantly associated with colorectal cancer risk (OR 1.14, 95% CI 0.63–2.10). Diabetic patients using insulin had an increased risk of colorectal cancer as compared with those not using it (OR 2.20, 95% CI 1.12–4.33); the OR increased with increasing duration of insulin use and was 8.18 (95% CI 2.06–32.50) for ≥10 years of use (*p*-value for trend 0.002). Risk estimates did not meaningfully change when we adjusted the models for duration of diabetes. When we mutually adjusted our estimates for insulin and metformin, the OR was 0.52 (95% CI 0.26–1.03) for metformin and 2.00 (95% CI 1.00–3.99) for insulin (data not shown). Using as reference category non-diabetic patients besides patients using other antidiabetic drugs, the OR was 1.11 (95% CI 0.82–1.53) for any oral medications, 0.98 (95% CI 0.69–1.40) for metformin, 1.18 (95% CI 0.75–1.86) for sulfonylurea derivatives, and 2.04 (95% CI 1.18–3.55) for insulin use, with significant trend in risk by increasing duration of insulin use (*p*-value for trend 0.003).

**Table 4:** **Distribution of 109 diabetic colorectal cancer cases and 135 diabetic controls, with corresponding odds ratios (ORs) and 95% confidence intervals (CIs), according to use of antidiabetic medications**.

Antidiabetic medications	Cases		Controls		OR^a^ (95% CI)
	*N*	(%)	*N*	(%)	
**Any oral antidiabetic drug**					
No^b^	9	(8.3)	7	(5.2)	1^c^
Yes	100	(91.7)	128	(94.8)	0.59 (0.18–1.92)
Duration (years)					
1 to <5	38	(38.0)	39	(30.5)	0.72 (0.20–2.50)
5 to <10	19	(19.0)	36	(28.1)	0.32 (0.08–1.25)
≥10	34	(34.0)	45	(35.2)	0.50 (0.14–1.78)
Missing	9	(9.0)	8	(6.3)	
*p*-Value for trend					0.21
**Metformin**					
No^d^	36	(33.0)	32	(23.7)	1^c^
Yes	73	(67.0)	103	(76.3)	0.47 (0.24–0.92)
Duration (years)					
1 to <5	34	(46.6)	34	(33.0)	0.74 (0.33–1.66)
5 to <10	11	(15.1)	27	(26.2)	0.25 (0.09–0.69)
≥10	20	(27.4)	34	(33.0)	0.36 (0.15–0.85)
Missing	8	(11.0)	8	(7.8)	
*p*-Value for trend					0.005
**Sulfonylurea derivatives**					
No^d^	68	(62.4)	82	(60.7)	1^c^
Yes	41	(37.6)	53	(39.3)	1.14 (0.63–2.10)
Duration (years)					
1 to <5	14	(34.2)	15	(28.3)	1.60 (0.62–4.09)
5 to <10	7	(17.1)	17	(32.1)	0.59 (0.21–1.64)
≥10	16	(39.0)	19	(35.9)	1.11 (0.47–2.61)
Missing	4	(9.8)	2	(3.8)	
*p*-Value for trend					0.92
**Insulin**					
No^d^	73	(67.0)	108	(80.0)	1^c^
Yes	36	(33.0)	27	(20.0)	2.20 (1.12–4.33)
Duration (years)					
1 to <5	15	(41.7)	16	(59.3)	1.52 (0.63–3.71)
5 to <10	5	(13.9)	4	(14.8)	1.86 (0.43–8.04)
≥10	14	(38.9)	4	(14.8)	8.18 (2.06–32.50)
Missing	2	(5.6)	3	(11.1)	
*p*-Value for trend					0.002

*Spain, 2007–2013*.

*^a^Estimates from multiple logistic regression models including terms for study center, sex, age, education, tobacco smoking, alcohol drinking, body mass index, physical activity, statin use, and aspirin use*.

*^b^This category includes patients using insulin*.

*^c^Reference category*.

*^d^This category includes also patients using other antidiabetic medications*.

As compared with diabetic patients using other antidiabetic medications, the OR of colorectal cancer was 0.69 (95% CI 0.37–1.28) for diabetic patients using metformin in monotherapy, 4.29 (95% CI 1.61–11.43) for those using sulfonylureas derivatives in monotherapy, and 1.70 (95% CI 0.52–5.51) for those using insulin in monotherapy; for combinations of therapies, the OR was 0.50 (95% CI 0.23–1.07) for diabetic patients using both metformin and sulfonylurea derivatives, 1.64 (95% CI 0.67–4.00) for those using both metformin and insulin, 0.97 (95% CI 0.19–4.91) for those using both sulfonylurea derivatives and insulin, and 3.41 (95% CI 0.56–20.71) for those using metformin, sulfonylurea derivatives, and insulin (Table S2 in Supplementary Material). Moreover, these estimates were consistent when we included in the reference category also non-diabetic patients. However, they should be interpreted cautiously since they were based on a small number of patients.

## Discussion

In our study, we find a 20% increased risk of colorectal cancer (though of borderline significance) in relation to type 2 diabetes, the excess risk being higher for a longer diabetes history and for proximal colon cancer. We provided further evidence of a possible inverse association with metformin and a positive association with insulin use, with significant duration–risk relationships.

Previous case–control and cohort studies showed an approximately 30% excess colorectal cancer risk in patients with diabetes as compared with those without diabetes (7–9). We found a somewhat lower association in our study which was, however, consistent in both the Italian and Spanish study. We found a higher risk of colorectal cancer for a history of diabetes of 10 or more years, although in the absence of a significant trend in risk. Similarly, a meta-analysis of six epidemiological studies reported no trend in risk for longer time since diabetes diagnosis (9). Furthermore, we found no differences by sex, again in agreement with the available evidence (7, 9, 13, 14). The 50% increased risk of proximal colon cancer is consistent with findings from previous studies reporting stronger associations with proximal than with distal colon cancer (7, 9, 21).

In the present study, we found a twofold excess risk of colorectal cancer in diabetic patients using insulin as compared with users of oral antidiabetic medications, with a significant increased risk with increasing duration of exposure. A similar increase was observed when the reference category also included non-diabetic patients and when we considered insulin use as monotherapy. This is consistent with the evidence from a meta-analysis − based on seven case–control studies and five cohort studies published before 2014 − which reported an overall RR of colorectal cancer of 1.69 (95% CI 1.25–2.27) for insulin users (22). However, the results of subsequent cohort studies were somewhat inconsistent (23, 24). Insulin has been shown to increase the risk of colorectal and other cancers by influencing the insulin-like growth factor system, which, in turn, may stimulate cellular proliferation and inhibit apoptosis (25). Insulin injection has also been shown to stimulate the growth of colorectal cancer precursors in animal models (26).

Conversely, metformin use was associated to a 50% reduced colorectal cancer risk as compared with use of other antidiabetic drugs, with a trend of decreasing risk with increasing duration of exposure. Consistently, a meta-analysis of five studies conducted up to 2011 reported an overall relative risk, RR, of 0.64 (95% CI 0.54–0.76) for metformin use (16). A weaker inverse association was reported in two subsequent meta-analyses that included at least nine epidemiological studies, with pooled RRs ranging between 0.55 and 0.90 depending on the reference category used (15, 17). Such inverse association was found in cohort studies (RR 0.82, 95% CI 0.71–0.94), but not in case–control studies (RR 1.04, 95% CI 0.94–1.15) (15). On the contrary, in a subsequent British cohort of patients with diabetes, metformin was not associated with colorectal cancer mortality (hazard ratio, HR, 1.06, 95% CI 0.80–1.40) (23). Metformin may reduce colorectal cancer risk by activating adenosine monophosphate-activated protein kinase and by consequent inhibition of the mammalian target of rapamycin (mTOR) (27). Moreover, it can suppress azoxymethane-induced formation of colorectal aberrant crypt foci (28, 29). A more widespread use of metformin as a first-line antidiabetic medication and its use in less severe cases of diabetes may also explain – at least in part – the lower association for history of diabetes observed in this study as compared with those conducted in 1980s and 1990s (10, 12). Moreover, in our study, the inverse relation with metformin was weaker when the reference category also included non-diabetic patients and in patients using metformin as monotherapy, suggesting that metformin may reduce the detrimental effect of insulin, rather than having a protective effect itself.

We found no consistent role of sulfonylurea derivatives on colorectal cancer risk, the OR being 1.14 when users of sulfonylureas were compared with diabetic patients using other antidiabetic medications and 1.18 when the reference category also included non-diabetic patients. Although we found a significant fourfold excess risk in diabetic patients using sulfonylurea derivatives as monotheraphy, this estimate was based on a small number of patients and should be cautiously interpreted. Similarly, a meta-analysis of three case–control and four cohort studies reported a colorectal cancer RR of 1.11 (95% CI 0.97–1.26) in users of sulfonylureas as compared with non-users (15). Similarly, in a subsequent cohort of patients with diabetes, sulfonylurea was not significantly associated with colorectal cancer mortality (HR 1.14, 95% CI 0.86–1.51) (23).

Limitations of this study are those typical of all case–control studies, including selection and information bias. However, in both countries, cases and controls came from comparable catchment areas, were interviewed by uniformly trained interviewers, and had a satisfactory participation rate for their study design. In particular, the high response rate in Italy reflects a traditionally high compliance of subjects approached for interview in the Italian case–control network; in addition, controls were hospital-based in Italy, while population-based in Spain, this explaining the higher response rates among controls in Italy as compared with Spain. Patients and interviewers were unaware of any hypothesis relating diabetes with colorectal cancer, thereby reducing the likelihood of potential selection and recall bias. Diagnosis of diabetes was self-reported, thus some potential misclassification is possible; however, the reliability of information on diabetes provided by hospital controls has been shown to be satisfactory (30) and the prevalence estimates of diabetes in our control groups were consistent with those from national population-based surveys (31, 32). Although in the Spanish study we could not distinguish between type 1 and type 2 diabetes, we excluded patients with a diagnosis of diabetes before age 30, who have been likely affected by type 1 diabetes. Another limitation is the lack of information on changes in medication type over time – which are frequent in diabetic patients – on glycemic level, and on the severity of the disease. Indeed, diabetic patients using insulin tend to have a more long-lasting and severe disease than non-insulin users. Among the strengths of our study is the allowance for major confounding factors for colorectal cancer, including body mass index and physical activity. Although in our dataset we did not have any information on household income and insurance, all estimates were adjusted for education, which has been shown to be a good proxy of socioeconomic status. Another strength of our study is that we were able to analyze the relation with duration of diabetes and use of various antidiabetic medications, exploring long-term exposures (i.e., >10 years), which have been examined in a limited number of studies.

Our study confirmed a positive association between diabetes and colorectal cancer, mainly proximal colon cancer. Among antidiabetic medications, it indicates a favorable effect for metformin use and a detrimental effect for insulin use, suggesting a need for enhanced screening measures for colorectal cancer in insulin-treated patients. It is, however, not easy to disentangle the effect of each antidiabetic medication since diabetic patients often use more than one medication, especially for more severe disease. Thus, it is difficult to establish whether these associations are causal or influenced by different severity of the disease and confounding.

## Author Contributions

VR took care of data management, statistical analysis, and manuscript draft; AT contributed in the result interpretation and revised the manuscript; EG-L and EG took care of data management for the Spanish center; GC-V, CV, MK, VM, JP, DS, FP, FB, CV contributed in the design of the HIWATE study and collection of the data; CB has the idea of the study and supervised the analysis and the manuscript drafting; she is the guarantor of this work and, as such, had full access to all the data in the study and takes responsibility for the integrity of the data and the accuracy of the data analysis. All the authors revised the final manuscript.

## Conflict of Interest Statement

The authors declare that the research was conducted in the absence of any commercial or financial relationships that could be construed as a potential conflict of interest.
