# Supplementary material for: Type 2 Diabetes, Antidiabetic Medications, and Colorectal Cancer Risk: Two Case–Control Studies from Italy and Spain
Source: Front Oncol. 2016 Oct 6;6:210. doi: 10.3389/fonc.2016.00210 (PMC5052265; doi:10.3389/fonc.2016.00210)
Supplement: Supplementary file 1 [file Table_1.DOC]

**Supplementary Table 1.** Distribution of 1,147 colorectal cancer cases and 1,594 controls, with corresponding odds ratios (ORs) and 95% confidence intervals (CIs), according to history of diabetes in strata of selected covariates. Italy and Spain, 2007-2013.

| Covariates | History of diabetes | | OR^a^ (95% CI) | p-value for heterogeneity^b^ |
| --- | --- | --- | --- | --- |
|  | No | Yes |  |  |
|  | Cases/  controls | Cases/  controls |  |  |
|  |  |  |  |  |
| Sex |  |  |  |  |
| Men | 641/863 | 109/135 | 1.13 (0.85-1.52) |  |
| Women | 347/543 | 50/53 | 1.49 (0.94-2.36) | 0.46 |
| Age |  |  |  |  |
| < 65 | 403/642 | 35/52 | 1.10 (0.67-1.80) |  |
| ≥ 65 | 585/764 | 124/136 | 1.22 (0.92-1.62) | 0.61 |
| Education (years) |  |  |  |  |
| < 8 | 561/668 | 115/122 | 1.15 (0.85-1.55) |  |
| ≥ 8 | 425/737 | 44/66 | 1.32 (0.85-2.05) | 0.87 |
| Missing | 2/1 | 0/0 |  |  |
| Tobacco smoking |  |  |  |  |
| Never smokers | 398/600 | 61/64 | 1.40 (0.93-2.12) |  |
| Ever smokers | 584/804 | 97/124 | 1.13 (0.83-1.54) | 0.54 |
| Missing | 6/2 | 1/0 |  |  |
| Alcohol consumption (drink/day) |  |  |  |  |
| < 1 | 455/701 | 90/99 | 1.34 (0.96-1.89) |  |
| ≥ 1 | 488/605 | 54/72 | 0.95 (0.64-1.42) | 0.09 |
| Missing | 45/100 | 15/17 |  |  |
| Body mass index (kg/m^2^) |  |  |  |  |
| < 25 | 356/508 | 43/36 | 1.77 (1.07-2.94) |  |
| ≥ 25 | 631/894 | 116/152 | 1.05 (0.79-1.40) | 0.21 |
| Missing | 1/4 | 0/0 |  |  |
| Physical activity (METS h/week) |  |  |  |  |
| ≤ 8 | 575/783 | 100/125 | 1.04 (0.76-1.42) |  |
| > 8 | 413/586 | 59/62 | 1.41 (0.93-2.15) | 0.43 |
| Missing | 0/37 | 0/1 |  |  |
| Statin use |  |  |  |  |
| Never | 813/1056 | 96/88 | 1.21 (0.88-1.67) |  |
| Ever | 170/348 | 63/100 | 1.14 (0.77-1.69) | 0.83 |
| Missing | 5/2 | 0/0 |  |  |
| Regular aspirin use |  |  |  |  |
| Never | 858/1204 | 119/132 | 1.18 (0.89-1.57) |  |
| Ever | 119/195 | 38/55 | 1.29 (0.76-2.21) | 0.69 |
| Missing | 11/7 | 2/1 |  |  |

METS: metabolic equivalents of task.

^a^ Estimates from multiple logistic regression models including terms for study centre, sex, age, education, tobacco smoking, alcohol drinking, body mass index, physical activity, statin use, and aspirin use. Reference category: no history of diabetes. ^b^ Heterogeneity between strata of the covariates was based on likelihood ratio tests and the resulting χ^2^ statistics.
