# Supplementary material for: Type 2 Diabetes, Antidiabetic Medications, and Colorectal Cancer Risk: Two Case–Control Studies from Italy and Spain
Source: Front Oncol. 2016 Oct 6;6:210. doi: 10.3389/fonc.2016.00210 (PMC5052265; doi:10.3389/fonc.2016.00210)
Supplement: Supplementary file 2 [file Table_2.DOC]

**Supplementary Table 2.** Distribution of 109 diabetic colorectal cancer cases and 135 diabetic controls, and corresponding odds ratios (ORs) and 95% confidence intervals (CIs), according to use of antidiabetic medications in monotherapy or in combinations. Spain, 2007-2013.

| Antidiabetic medications | Cases | | Controls | | OR^a^ (95% CI) |
| --- | --- | --- | --- | --- | --- |
|  | N | (%) | N | (%) |  |
|  |  |  |  |  |  |
| Metformin monotherapy |  |  |  |  |  |
| No ^b^ | 72 | (66.1) | 84 | (62.2) | 1^c^ |
| Yes | 37 | (33.9) | 51 | (37.8) | 0.69 (0.37-1.28) |
| Sulfonylurea derivatives monotherapy |  |  |  |  |  |
| No ^b^ | 91 | (83.5) | 126 | (93.3) | 1^c^ |
| Yes | 18 | (16.5) | 9 | (6.7) | 4.29 (1.61-11.43) |
| Insulin monotherapy |  |  |  |  |  |
| No ^b^ | 100 | (91.7) | 128 | (94.8) | 1^c^ |
| Yes | 9 | (8.3) | 7 | (5.2) | 1.70 (0.52-5.51) |
| Metformin and sulfonylureas derivatives |  |  |  |  |  |
| No ^b^ | 95 | (87.2) | 103 | (76.3) | 1^c^ |
| Yes | 14 | (12.8) | 32 | (23.7) | 0.50 (0.23-1.07) |
| Metformin and insulin |  |  |  |  |  |
| No ^b^ | 93 | (85.3) | 122 | (90.4) | 1^c^ |
| Yes | 16 | (14.7) | 13 | (9.6) | 1.64 (0.67-4.00) |
| Sulfonylureas and insulin |  |  |  |  |  |
| No ^b^ | 105 | (96.3) | 130 | (96.3) | 1^c^ |
| Yes | 4 | (3.7) | 5 | (3.7) | 0.97 (0.19-4.91) |
| Metformin, sulfonylureas and insulin |  |  |  |  |  |
| No ^b^ | 104 | (95.4) | 133 | (98.5) | 1^c^ |
| Yes | 5 | (4.6) | 2 | (1.5) | 3.41 (0.56-20.71) |
|  |  |  |  |  |  |

^a^ Estimates from multiple logistic regression models including terms for study centre, sex, age, education, tobacco smoking, alcohol drinking, body mass index, physical activity, statin use, and aspirin use. ^b^ This category includes also patients using other anti-diabetic medications. ^c^ Reference category.
